# Immune Checkpoint Dysregulation in Autoimmune Disorders: A Narrative Review of Therapeutic Implications

**DOI:** 10.7759/cureus.97002

**Published:** 2025-11-16

**Authors:** Diwan Mahmood Khan, Rohit Kumar, Mahavadi Sriharsha, Srihita Mahavadi, Arnaw Kishore

**Affiliations:** 1 Department of Microbiology, Varun Arjun Medical College & Rohilkhand Hospital, Shahjahanpur, IND; 2 Department of Microbiology, Faculty of Medicine, Avalon University School of Medicine, Willemstad, CUW; 3 Department of Critical Care Medicine, Apollo Hospitals, Hyderabad, IND; 4 Department of Biochemistry, Asian Institute of Gastroenterology (AIG) Hospitals, Hyderabad, IND; 5 Department of Microbiology, East Point College of Medical Science, Bengaluru, IND

**Keywords:** autoimmune disorders, co-stimulation blockade, ctla-4, extracellular vesicles, immune checkpoints, pd-1/pd-l1, regulatory t cells

## Abstract

Immune checkpoints act like dimmer switches that keep immune responses in balance. In autoimmune disease, problems usually fall into four overlapping types: weak inhibitory signals, too much co-stimulation, metabolic or epigenetic rewiring of checkpoint pathways, and a mismatch between tissue and blood findings.

This narrative review focused on human and translational studies from PubMed/Medical Literature Analysis and Retrieval System Online (MEDLINE), Excerpta Medica database (Embase), Web of Science, and Scopus (to 2025), prioritizing tissue-based data, trials of checkpoint agonists or co-stimulation blockade, cell-based tolerance (low-dose interleukin-2 (IL-2), chimeric antigen receptor regulatory T cells (CAR-Tregs), and extracellular vesicle (EV) approaches.

Tissue profiling tracks disease activity better than blood alone. Immunometabolic stress, especially lactate-driven protein lactylation and ferroptosis, can blunt programmed cell death protein-1/cytotoxic T-lymphocyte associated protein-4 (PD-1/CTLA-4) braking and destabilize regulatory T cells (Tregs). A practical biomarker panel pairs lesion immunohistochemistry/spatial maps of PD-1/programmed death-ligand-1 (PD-L1) and second-wave checkpoints with soluble PD-1, PD-L1, CTLA-4, and EV cargo under strict pre-analytical control.

Therapy should be staged: first, lower the inflammatory and metabolic load, then restore inhibitory tone with checkpoint agonists or co-stimulation blockade, and add Treg support and EV-guided delivery when regulation is fragile. Safety needs vaccine timing, age-/sex-aware dosing, and composite panels that distinguish flare from over-suppression. The next step is endotype-first, spatially informed, biomarker-anchored trials to achieve durable, safer immune rebalancing.

## Introduction and background

Immune checkpoints act as rheostats-integrating antigen strength, co-signals, cytokines, and tissue context to calibrate activation, differentiation, contraction, and restoration of homeostasis. Inhibitory axes, programmed cell death protein 1/programmed death-ligand 1 (PD-1/PD-L1), cytotoxic T-lymphocyte-associated protein 4 (CTLA-4), lymphocyte activation gene-3 (LAG-3), T-cell immunoglobulin and mucin-domain containing-3 (TIM-3), T-cell immunoreceptor with immunoglobulin and immunoreceptor tyrosine-based inhibitory motif domains (TIGIT), B and T lymphocyte attenuator (BTLA), V-domain immunoglobulin suppressor of T-cell activation (VISTA), attenuate proximal and distal signalling to restrain effector programmes and preserve self-tolerance, while co-stimulatory pathways (CD28, inducible T-cell co-stimulator (ICOS), OX40/CD134, 4-1BB/CD137, CD40) amplify priming, metabolic fitness, survival, and lineage commitment. These are graded controls, not binary switches, shaping response quality across lymphoid organs, blood, and tissues [1,2]. In autoimmunity, “dysregulation” typically reflects four overlapping patterns: (i) insufficient inhibitory tone, (ii) excessive co-stimulation, (iii) metabolic/epigenetic decoupling of checkpoint signals, and (iv) compartmental mismatch between tissue, blood, and lymphoid niches, each with implications for patient stratification and therapy design [1,3,4]

Immune checkpoints as guardians of self-tolerance

Insufficient Inhibitory Tone

Diminished PD-1/PD-L1 and CTLA-4 activity sustains T-cell receptor (TCR) signalling, co-stimulation, and pathogenic cytokines. Lesion-level data show this directly: in immunoglobulin G4 (IgG4)-related dacryoadenitis, altered PD-(L)1 landscapes correlate with clinical activity and treatment response, indicating failure of local inhibitory brakes. In cutaneous autoimmunity, quantitative/functional regulatory T-cell (Treg) insufficiency aligns with loss of CTLA-4-coupled suppression. Briefly, sex-specific modulation can tilt these brakes: estrogen has been reported to increase PD-L1 expression, potentially recalibrating PD-1 signalling and Treg fitness/exhaustion in inflamed tissues, consistent with evidence that checkpoint tone in Tregs is context-dependent and shaped by extrinsic cues [5-7].

Excessive Co-stimulation

Persistent CD28/ICOS/OX40/4-1BB/CD40 signalling expands autoreactive clonotypes. In rheumatoid arthritis (RA), T peripheral helper (Tph) and related helper states deliver sustained B-cell help in synovium when augmented co-stimulation overwhelms exhausted or numerically constrained inhibitory circuits, paralleling PD-1 high T-follicular helper/T-follicular regulatory (Tfh/Tfr) imbalance in follicles [1,3,8].

Metabolic/Epigenetic Decoupling

Checkpoint function is tuned by immunometabolism and chromatin state. In psoriasis, lactate accumulation and protein lactylation reprogram transcription in keratinocytes and immune cells, stabilizing inflammatory circuits that blunt canonical inhibitory signalling [9]. Ferroptosis, iron-dependent lipid peroxidation, interfaces with nuclear factor-kappa B (NF-κB), Janus kinase, signal transducer and activator of transcription (JAK-STAT), and the mechanistic target of rapamycin (mTOR), reshaping antigenicity and T-cell fate and offering routes by which inflammatory metabolism uncouples checkpoints from tolerance enforcement [10]. Treg identity and suppression are likewise metabolically programmable; bioenergetic shifts erode checkpoint-coupled Treg control [11].

Compartmental Mismatch

Circulating checkpoint states often diverge from those in lesions. In IgG4-related disease (IgG4-RD), lesion PD-L1 and cellular neighborhoods track activity better than blood readouts, underscoring tissue-anchored targeting [7]. Sex and hormonal context further modulate checkpoint biology across compartments [3,12]. Extracellular vesicles (EVs) add an inter-compartment layer: vesicles ferry PD-L1 and regulatory cargo that echo local metabolic tone, enabling liquid snapshots of tissue “inhibitory tone” [13,14].

Together, loss of braking, excess “gas,” metabolic/epigenetic decoupling, and compartment mismatch offer a practical lens to interpret human datasets and anticipate why checkpoint-modulating therapies succeed or fail across patients and tissues [1,5,7].

Why a narrative review now?

Human Tissue Clarity

High-content lesion profiling links checkpoint expression/function to disease behavior, moving beyond blood-only heuristics. Lactate-driven lactylation links glycolysis to Th17/Tc17 tilt and Treg fragility, while ferroptosis can lift PD-L1 and reshape antigen display. However, readouts are context-sensitive (lactate/lactyl marks, GPX4-ALOX axes), EV assays vary by isolation, and several human datasets are underpowered, e.g., IgG4-RD tissue cohorts with n<50 and subgroups in single digits, so claims remain hypothesis-generating. The path forward is multicenter, longitudinal validation with tight pre-analytics and integrated spatial-soluble profiling [7]. In rheumatic disease, Tph/Tfh/Treg wiring explains tissue damage trajectories [1]. Quantitative syntheses consolidate Treg defects in skin autoimmunity [5].

Immunometabolism Breakthroughs

Lactate/lactylation regulates inflammatory transcription and can explain checkpoint non-response in glycolytic niches [9]. Ferroptosis couples lipid peroxidation and iron handling to antigenicity and cytokine circuits, motivating metabolic checkpoint combinations [10]. Treg bioenergetics ties metabolic repair to restored checkpoint-coupled suppression [2,11].

Therapeutic Pivot

Oncology’s checkpoint blockade validates the centrality of inhibitory brakes and teaches immune-related adverse event (irAE) guardrails [15-17]. Autoimmunity is exploring the converse, checkpoint agonism/co-stimulation dampening, alongside chimeric antigen receptor (CAR) T cells and CAR regulatory T cells (CAR-Tregs) for antigen-specific control and vesicle-based delivery to re-educate niches [18].

The aim of synthesis is (i) tissue-anchored biology across compartments [1,7]; (ii) metabolic and cell-death programmes that decouple checkpoints [9-11]; and (iii) a maturing pipeline of checkpoint agonists, CAR-Tregs, and exosome therapeutics framed by oncology-derived safety lessons [13,15,16,18,19]. The goal is a checkpoint-centric framework for target selection, patient stratification, and longitudinal monitoring.

## Review

Database selection

We conducted a structured narrative search (PubMed/Medical Literature Analysis and Retrieval System Online (MEDLINE), Excerpta Medica database (Embase), Web of Science, and Scopus; through 2025). using controlled vocabulary and keywords for checkpoints and autoimmunity programmed cell death protein-1 (PD-1)/programmed death-ligand-1 (PD-L1) and cytotoxic T-lymphocyte-associated protein-4 (CTLA-4)/LAG-3/TIM-3/TIGIT/BTLA/VISTA; CD28/ICOS/OX40/4-1BB/CD40; Treg/Tfh; lactate/lactylation/ferroptosis; exosomes/EVs; CAR-Treg; checkpoint agonist).

Filters used were English; human studies prioritized; pivotal mechanistic/animal work included when directly informative. We excluded conference abstracts, editorials without data, single-case reports (unless mechanistically novel), and non-peer-reviewed preprints. Two reviewers screened titles/abstracts and full texts; disagreements were resolved by consensus. We complemented database retrieval with citation chasing and hand searches. Priorities were as follows: human tissue studies, translational work, interventional trials (agonists/modulators, CAR-Tregs, vesicle therapies), and high-quality syntheses. Data were extracted into a standardized template. The aim was mechanistic depth rather than exhaustive enumeration.

Biology of immune checkpoints in self-tolerance

Core Inhibitory Axes

PD-1/PD-L1/programmed death-ligand-2 (PD-L2) and CTLA-4 provide first-line braking of self-reactivity, with PD-1 engagement muting proximal T-cell activation to preserve peripheral tolerance, while CTLA-4 competes with CD28 for CD80/CD86 and removes these ligands from antigen-presenting cells through trans-endocytosis to stabilize regulatory control. Secondary inhibitory receptors, LAG-3, TIM-3, TIGIT, BTLA, and VISTA, often co-express with PD-1 and collectively dampen T-follicular help and cytotoxic drive; insufficiency across this layer is repeatedly linked to autoimmune flare biology and is drawing interest for measured co-targeting strategies. Fluid-phase readouts connect mechanism to monitoring: soluble PD-1/PD-L1 and extracellular-vesicle PD-L1 mirror activation/exhaustion states and show translational promise for dynamic disease assessment and targeted delivery in systemic autoimmune disorders. Clinically, the CTLA-4 axis already translates abatacept and demonstrates signal-2 dampening in RA and at-risk states, while structural/trafficking data on CTLA-4 reinforce how ligand competition and surface cycling underwrite this effect. Stepping back, a consistent picture emerges across settings: chronic inflammatory tone reshapes checkpoint expression and function in both cancer and autoimmunity, but in opposite directions, making PD-1/CTLA-4 the foundation and the second-wave receptors the fine-tuning needed to restore a durable, safer balance [4,13,20,21].

Co-stimulatory Circuits

CD28 licenses priming; ICOS sustains follicular help; OX40 and 4-1BB extend effector survival; and CD40L-CD40 sets germinal center (GC) tempo and class switching. When this circuitry runs hot, Tfh/Tph-B loops escalate and ectopic GCs appear, notably in Sjögren’s. Clinical signal backs the axis: CD40L antagonism with dazodalibep improved systemic activity and symptoms in a phase-2 Sjögren’s trial, while broad co-stimulation dampening (CTLA-4-Ig) remains a benchmark in RA. Strategy in one line: lower global co-stimulation where needed, or bias toward regulatory paths (tilting ICOS toward Tfr rather than Tfh) to quiet GC output without flattening host defence [8].

Cellular Ecology: Tregs

FOXP3⁺ Tregs maintain lineage stability through tonic IL-2/CD25 signalling while balancing mTOR-FOXO-guided metabolic programs; perturbing this axis (for example, via mTORC1/2-FOXO1 shifts) undermines CD25/CTLA-4 expression and tissue homing, predisposing to dysfunction. In inflamed niches, glycolysis, hypoxia, and lactate accumulation reshape immunity; lactate-driven lysine lactylation rewires gene expression and amplifies IL-17-skewed circuits, a pattern linked to psoriasis severity and relapse biology. Clinically, IL-2-based Treg support shows promise yet mixed signals: in systemic lupus erythematosus (SLE), Tregs display exhaustion-like programs and type-I-IFN-driven coinhibitory networks that blunt function, helping explain the variable efficacy of standalone low-dose IL-2 and arguing for combinations that also ease metabolic stress. Reinforcing the disease-specific context, a 2025 meta-analysis in dermatology quantifies Treg deficits and highlights that IL-2/Treg-inducing approaches remain preliminary and heterogeneous across studies. By contrast with “fragile” Tregs in glycolytic, lactate-dense psoriatic skin, rheumatoid synovium often contains more Tregs with intact in-vitro suppression but restrained proliferation and a differentiated effector-Treg phenotype, “functionally constrained” rather than absent. Extracellular vesicles further tune this ecology; disease-skewed EV cargo in SLE/RA can inhibit Tregs or promote interferon-rich loops, suggesting that responsiveness to IL-2 depends on broader intercellular signalling. Finally, pairing IL-2 with metabolic modulators that bolster Tregs (e.g., mTOR pathway inhibitors) is mechanistically supported across autoimmune settings and may be required to “keep suppressors fit, then tune the brakes” [2,5,6,9-11,13,22,22].

Cellular Ecology: Tfh/Tph and B Cells

PD-1 and ICOS gradients shape Tfh maturation and Tfr restraint inside GCs. Disturbed ratios (↑Tfh, ↓Tfr) track with autoantibody pressure and disease activity; ICOS and CD40L sit on that same stem, guiding help and control inside the follicle. In inflamed niches, high-PD-1 states expand and reflect chronic stimulation; across chronic inflammatory settings, exhausted PD-1+ profiles recur. The monitoring lever is simple: restore the Tfh/Tfr balance, and GC noise falls [3,23].

Cellular Ecology: Myeloid/Innate and EV Layer

Dendritic cells and macrophages gate T-cell tone through ligand display and through what they shed. Extracellular vesicles (from dendritic cells (DCs), Tregs, mesenchymal stromal cells (MSCs), and neutrophils) carry proteins, lipids, and RNAs that can rewrite local signalling, shaping antigen presentation, cytokines, and differentiation. This vesicular layer gives a gentler dial on tolerance, and it is engineerable: targeted EVs can deliver checkpoint cues or tolerogenic cargo to inflamed tissue. Microenvironmental stress (hypoxia, nutrients) further nudges these programs, linking metabolism to ligand density and EV output [11,22].

Cross-cutting mechanisms that rewire checkpoints

Immunometabolism and Epigenetics

Lactate & lactylation: Glycolytic, lactate-rich niches drive histone lactylation that tilts transcription toward Th17/Tc17 programs, weakens dendritic-cell performance, and erodes Treg stability conditions under which PD-1/CTLA-4 signalling loses “grip.” Targetable rungs include transport (MCT1/4), LDH-A, and the lactylation writers/erasers themselves. A parallel conduit is vesicular: EV cargo mirrors metabolic stress and disseminates these checkpoint cues across tissues. Biomarkers to validate in accessible matrices: plasma/serum lactate, LDH activity, and EV checkpoints (e.g., EV-PD-L1) alongside lactyl-histone marks in tissue, where feasible. Pre-analytics matter (prompt processing on ice; RBC-free plasma for lactate; EV isolation/QC) [9,13].

mTOR-HIF-1α-glycolysis: Effector bias rides on mTORC1 and HIF-1α. Ordinarily, PD-1 dampens PI3K-Akt-mTOR and nudges cells toward fatty-acid oxidation, but persistent antigen or hypoxia lets HIF-1α dominate and bypass PD-1 braking. Therapeutic sequence: reduce metabolic pressure (mTOR/HIF-1α/lactate) first, then layer checkpoint agonism so inhibitory tone can hold [2].

Ferroptosis: Lipid peroxidation releases DAMPs (HMGB1, ATP), primes NF-κB/inflammasomes, and can upregulate PD-L1 while skewing T-cell metabolism. Time matters: early ferroptosis can mature DCs; late ferroptosis fuels immune escape. Clinical readouts: 4-HNE adducts, ACSL4, GPX4/LOX axes, plus soluble/EV checkpoint panels. Intervention cue: blunt ferroptotic injury (without stripping antimicrobial defence) and pair with checkpoint repair to prevent rebound inflammation [10,22].

NF-κB / JAK-STAT: These pathways induce checkpoint ligands yet simultaneously fuel glycolysis and co-stimulation, explaining the familiar paradox of high PD-L1 alongside effector dominance. Overactive STAT3/NF-κB erodes FOXP3 stability and undermines Treg control. So, the sequence matters: quiet JAK/NF-κB pressure, rebalance metabolism, then restore inhibitory tone via PD-1/CTLA-4 agonism in suitable endotypes [5,6].

EVs, miRNAs, and engineered delivery: EVs ferry PD-L1 and immunoregulatory miRNAs; engineered vesicles and liposomes can be tuned to deliver tolerogenic cargo, conceptually supporting metabolic/epigenetic reprogramming (e.g., for Treg stability or to reset inflammatory tone) in checkpoint-brittle niches. Caveats & limitations. (i) Tissue-blood discordance: PD-L1 positivity does not guarantee inhibitory function; co-stimulation, cytokines, and metabolic wiring can override the brake. (ii) EV analytics vary by isolation method; harmonize protocols and include recovery/hemolysis indices in methods. (iii) Lactate is labile; draw-to-freeze time and anticoagulant choice shift values. (iv) Context-specificity: ferroptosis markers (e.g., 4-HNE) and checkpoint panels show disease-stage effects; interpret longitudinally and, where possible, pair with spatial tissue readouts [2,5,6,9,10,13,22].

Host Modifiers

Immunosenescence: With age, naïve T- and B-cell pools contract, low-grade inflammation rises, and PD-1hi multi-checkpoint phenotypes become common. Metabolic wiring shifts toward glycolysis and oxidative stress, so inhibitory signals register but don’t bite as hard. This combination changes the risk-benefit math for checkpoint-targeted strategies and argues for dose, schedule, and vaccine timing that are age-aware. Inflammatory milieus that already enrich for exhausted PD-1+ subsets illustrate the same pattern: brakes present, traction poor; hence, the need to reduce background cytokine/metabolic load before turning up inhibitory cues [2,17].

Sex differences: Estrogens boost Tfh quality and humoral amplitude; androgens tend to stabilize Tregs. Add X-linked dosage and hormone crosstalk with mTOR/JAK-STAT, and the net effect is a lower checkpoint threshold in many females and a higher bar in many males. That has practical read-through: sex-informed dose finding, cut-offs for soluble markers, and context on what “high PD-L1” means in a hormone-tuned niche. Even at the pathway level, hypoxia/HIF nodes can gate Th17-to-Treg balance in a sex-skewed fashion, reinforcing why stratification cannot be sex-blind [3,12].

Synthesis: Background biology sets the floor. Metabolic pressure (lactate/lactylation, ferroptosis) and inflammatory drivers (NF-κB/JAK-STAT) hard-wire effector programs and fray FOXP3 stability; age and sex then shift the threshold at which checkpoints work. The manuscript’s sequence holds: first, lower inflammatory and metabolic load; then, restore inhibitory tone with PD-1/CTLA-4 agonism or calibrated co-stimulation dampening; finally, deliver and monitor with tissue-anchored maps plus soluble and EV panels. EVs are not just readouts; engineered vesicles can carry tolerogenic cargo or checkpoint agonists into inflamed niches, while standardization and targeting remain active workstreams [2,3,9,10].

Disease-specific patterns of checkpoint dysregulation

SLE

A brittle Treg compartment meets an overdriven Tfh program, feeding plasmablast waves and high-affinity autoantibodies. PD-1hi signatures are present yet muted by cytokine flux and glucose-hungry metabolism, so inhibition fails to grip; ectopic GCs are frequent and stubborn. Practical signals include soluble PD-1/PD-L1, exosomal cargo bearing immune transcripts or ligands, and IL-21/Tfh modules that mirror GC heat. Therapy maps follow the sequence used across this review: first, calm the terrain (low-dose IL-2 to stabilize Tregs and control inflammation), then add targeted brakes. CTLA-4-Ig shows modest benefit; B-cell depletion or B-cell activating factor (BAFF)/a proliferation-inducing ligand (APRIL)-axis tactics carry the backbone; concept lanes PD-1 agonism, gentle ICOS-IL-21 dampening, anti-CD19 or BCMA CAR-T, and early CAR-Tregs aim to switch off GC noise without flattening host defence [3,23].

RA

The synovium is a low-oxygen, glycolytic niche. Myeloid cells can express PD-L1, but not enough and too late; co-stimulation fires early, while T peripheral helper (Tph) B-cell circuits keep the loop running. Lipid peroxidation and ferroptotic stress add to tissue injury and magnify NF-κB tone. Readouts are concrete: spatial maps of PD-(L)1 in synovium, soluble checkpoint panels, EV signatures, and oxidized lipid markers. Care follows a layered plan: CTLA-4-Ig remains established; tolerogenic dendritic cell strategies and PD-1 agonists are under evaluation; and metabolic co-therapy (mTOR/HIF-1α, lactate handling, and iron balance) is used to make inhibitory cues “bite” again and to reset the threshold at which checkpoints work [2,8].

Sjögren’s syndrome (SS)

Salivary glands host ectopic GCs fed by strong Tfh/Tph help. PD-1/PD-L1 landscapes are patchy; Tregs show fragility, so B-cell activation persists despite visible brakes. Biomarkers line up with the gland: PD-L1 immunohistochemistry on biopsies, salivary or exosomal miRNAs that track lympho-epithelial activity, and soluble checkpoint readouts. Management reflects this biology: B-cell depletion is central, IL-21/Tfh modulation tunes the fuel, and checkpoint agonism is a developing option. Mesenchymal stromal cell or exosome approaches seek to re-educate the exocrine niche. Importantly, CD40L antagonism provides a direct GC “off-switch” and fits the disease geometry [2,3].

Psoriasis/Psoriatic Arthritis (PsA)

The axis is metabolic first, then immune. IL-23 and then Th17 dominance sit on a lactate-rich base; lysine lactylation pushes transcription toward effector programs, loosens Treg grip, and blunts PD-1/CTLA-4 braking. Ferroptosis adds reactive lipids and strengthens NF-κB loops. Trackers are tangible: lactate/lactylation indices, ferroptosis-linked lipids (e.g., 4-HNE), and vesicle cargo tied to keratinocyte stress. Therapy starts with known biologics against IL-17/23/TNF. Next steps, in selected endotypes, include LDH/MCT inhibition to lower lactate pressure and cautious checkpoint calming where Tregs are fragile. Fix the fuel, then tame the flame [9].

Multiple Sclerosis (MS)

Entry to the CNS is gated. Microglia and border-zone myeloid cells display PD-L1, galectin-9 (Gal-9), CD155, and herpesvirus entry mediator (HVEM), shaping who gets in and who survives. Cyclic GMP-AMP synthase stimulator of interferon genes (cGAS-STING) tone and interferons remodel checkpoint display and alter trafficking across the blood-brain barrier. Biomarkers should therefore straddle compartments: CSF-soluble checkpoints plus imaging-genomic layers and EV profiling. Interventions favor antigen-coupled tolerance as the opener, followed by cytokine-pathway control; PD-1/CTLA-4 agonism, if used, should be sequenced carefully so gates are ready to hold. In short, prepare the doorway before pushing the brake [2,12,24].

Type 1 Diabetes (T1D)

β-cells under stress raise PD-L1, yet cytotoxic attack continues when co-stimulation and inflammation stay high. Treg support is thin, so tolerance lapses. Monitoring sets are simple and informative: islet autoantibodies, sPD-1/sPD-L1, β-cell-derived EVs, and Treg metrics. Therapy follows the same choreography: lower inflammatory tone, then restore restraint: antigen-specific tolerance platforms, low-dose IL-2 for regulatory maintenance, and islet-directed CAR-Tregs as an emerging precision lever. Protect the islet first; fine-tune checkpoints when the noise falls [11,12].

Inflammatory Bowel Disease (IBD)

The mucosa integrates microbial, nutrient, and hypoxic cues. Epithelial and myeloid ligands set the local threshold, yet Treg function commonly sags. Lactate pockets and ferroptotic epithelial injury activate NF-κB/JAK-STAT and reshape checkpoint density along the crypt luminal axis. Endotyping is practical: mucosal PD-L1 maps, stool EV profiles, and ferroptosis markers. Treatment logic is staged: build on anti-cytokine or anti-integrin backbones, add metabolic conditioning (reduce lactate load, protect from lipid peroxidation), and only then trial PD-1/CTLA-4 agonists in subsets where brakes can actually hold [9,10].

Anti-neutrophil Cytoplasmic Antibody (ANCA), Associated Vasculitis (AAV)

The milieu is NET-heavy and oxidant-rich. Effector T cells co-express multiple inhibitory receptors and still injure vessels because signals are distorted by redox stress and protease debris; vascular myeloid PD-(L)1 axes are altered and inconsistent. Biomarkers span soluble checkpoints, neutrophil extracellular trap (NET) components, and vesicle cargo from neutrophils and endothelium. Clinical care stays with induction and maintenance as the base. In remission, steroid-sparing options, PD-1 agonism at low doses, or IL-2 support for Tregs are concept-stage tools meant to keep the lid on without suppressing host defence [4].

IgG4-RD

Lesions are fibro-inflammatory and compartmentalised. PD-L1+ gradients in myeloid and dendritic cells are evident; tissues hold many Tregs but with skewed function, and GC-like structures are common in affected organs. Blood and tissue can diverge, so spatial profiling matters. Biomarker sets pair PD-L1 maps with sPD-L1, total IgG4, and EV cargo. Therapy uses steroids and rituximab as anchors; in fibrotic or metabolically stressed niches, consider PD-1/PD-L1 modulation together with anti-fibrotic and immunometabolic co-therapy to reset tone and slow scarring [7,12].

Vitiligo

Autoreactive CD8+ T cells target melanocytes, while the Treg pool is small and FOXP3 stability wavers. Metabolic stress in skin narrows checkpoint traction. Signals are accessible: Treg metrics, IL-17/IL-22 levels, soluble checkpoints, and EVs carrying melanocyte antigens. Management blends repigmentation with regulatory support, low-dose IL-2, careful checkpoint calming in select cases, and metabolic aid to protect Tregs. Guard the pigment bed; make space for tolerance to return (Table 1) [5,25].

**Table 1:** Evidence map of checkpoint dysregulation across autoimmune diseases Operationalises four dysregulation patterns into scannable, disease-level hypotheses that the rest of the review then tests. The “Tissue vs blood readouts” and “Candidate biomarkers” columns feed directly into Biomarkers & Platforms: lesion immunohistochemistry (IHC)/immunofluorescence (IF) for PD-(L)1 and second-wave checkpoints with spatial context; paired soluble panels (sPD-1/sPD-L1/sCTLA-4) with strict pre-analytical control; and EV signatures that mirror checkpoint tone and metabolic state. In line with the “compartmental mismatch” theme, we highlight that tissue positivity for PD-L1 does not guarantee functional braking in glycolytic or cytokine-rich niches, necessitating spatial-omics and multi-omics anchors rather than blood-only heuristics. APC: antigen-presenting cell; EV: extracellular vesicle; GC: germinal center; NET: neutrophil extracellular trap; RAPs: resistance-associated proteins; Tfh/Tph/Tfr: T-follicular helper/peripheral helper/regulatory; Kla: lysine lactylation

Study	Disease	Dominant pattern(s)	Tissue vs. blood readouts	Candidate biomarkers (tissue/soluble/EV)	Therapeutic levers (checkpoint agonists/co-stim blockers; cell-based; metabolic co-therapy)
Lu 2025; Yang 2025; Chen 2025 [2,11,18]	Systemic lupus erythematosus	Insufficient inhibitory tone (Treg instability); excessive co-stimulation (Tfh/Tph); compartment mismatch; STAT3/NF-κB pressure	Tissue: kidney/skin GC-like aggregates; PD-1^hi^ Tfh/Tph; spatial PD-L1 heterogeneity. Blood: sPD-1/sPD-L1/sCTLA-4; IL-21; EV miRNAs	Tissue PD-L1/ICOS-L; sPD-1/sPD-L1/sCTLA-4; IL-21; EV miR panels linked to B-cell activation	CTLA-4-Ig (modest); low-dose IL-2; B-cell reset (CD19/BCMA CAR-T); conceptual PD-1 agonism / ICOS dampening; engineered EVs
St Clair 2024; Shen 2025; Harel 2025; Chen 2025 [2,8,10,15]	Rheumatoid arthritis (RA)	Excessive co-stimulation (CD28/ICOS/OX40/Tph →B); insufficient inhibitory tone on myeloid; ferroptosis-linked stress	Tissue: hypoxic/glycolytic synovium; inducible but inadequate PD-L1 on Antigen-presenting cells (APCs). Blood: soluble checkpoints; proteomic RAPs	Synovial PD-L1 maps; 4-HNE/oxidized lipids; sPD-1/sPD-L1; EV PD-L1	CTLA-4-Ig established; PD-1 agonist (early RA niche); tolerogenic DCs; ferroptosis/lactate modulation to restore inhibition
Lv 2025; Life-Tfh 2025 [13,22]	Sjögren’s syndrome	Tfh/Tph dominance; Treg fragility; compartment mismatch (gland vs blood)	Tissue: salivary/lacrimal PD-L1 IHC; ectopic GCs. Fluids: saliva EVs; serum soluble	Gland PD-L1/ICOS-L; IL-21; EV miRNA/protein signatures	B-cell depletion; IL-21/Tfh modulation; concept-stage PD-1 agonism; MSC/engineered exosome therapy
Wu 2025; Shen 2025; Lerner 2025 [6,9,10]	Psoriasis/psoriatic arthritis	Metabolic/epigenetic decoupling (lactate→lactylation); ferroptosis; Treg insufficiency	Tissue: lesional Kla (lactylation), PD-(L)1 uncoupled from restraint. Blood: lactate/LDH; EV cargo	Histone/non-histone lactylation; LDH/MCT1/4; 4-HNE; EV PD-L1/miRNAs	IL-17/23/TNF biologics; LDH/MCT targeting; cautious checkpoint calming in Treg-fragile subset; Treg-support (low-dose IL-2)
Huang Z et al., 2025 [24]	Multiple sclerosis	Compartment mismatch (CNS vs blood); myeloid ligand gating (PD-L1, Gal-9, CD155, HVEM); cGAS–STING tone	Tissue: CNS myeloid checkpoints; BBB trafficking. CSF: soluble checkpoints; EVs	CSF sPD-L1/sGal-9; EV proteins/miRNAs; interferon/STING signatures	Antigen-coupled tolerance; sequence cytokine-pathway modulators with PD-1/CTLA-4 agonism (concept)
Lu 2025; Yang 2025 [11,18]	Type 1 diabetes	Insufficient inhibitory tone (Treg); strong co-stimulation; β-cell stress with PD-L1 upregulation, but override	Tissue: islet PD-L1; insulitis. Blood: autoantibodies + soluble checkpoints; Treg metrics	sPD-1/sPD-L1; islet-derived EVs; Treg frequency/function	Low-dose IL-2; antigen-specific tolerance; islet-homing CAR-Tregs (safety switches)
Shen 2025; Wu 2025 [9,23]	Inflammatory bowel disease	Treg impairment; metabolic decoupling (mTOR/HIF-1α, lactate); ferroptosis-linked epithelial injury	Tissue: mucosal PD-L1/second-wave checkpoints; ferroptosis markers. Stool/blood: EVs, lipid peroxidation	Mucosal PD-L1; glutathione peroxidase-4 (GPX4)” and “arachidonate lipoxygenase (ALOX); 4-HNE; EV signatures	Anti-cytokine/anti-integrin backbones + metabolic conditioning (mTOR/lactate/ferroptosis) to enable checkpoint agonism in endotypes
Li et al 2025; Chen 2025 [2,4]	ANCA-associated vasculitis	“Exhausted-yet-cytotoxic” effector T cells (multi-IR co-expression) within NET-osis-rich milieu; altered vascular APC PD-(L)1	Tissue: vessel wall ligand shifts; NETs. Blood: sPD-1/sPD-L1/sCTLA-4; MPO/PR3-ANCA; EVs	Soluble checkpoints; NET components; EV miRNAs	Standard induction/maintenance; concept-stage PD-1 agonism as steroid-sparing; IL-2 Treg support in remission
Bae 2025; Kim 2025 [5,12]	IgG4-related disease	Compartment mismatch; PD-L1^+^ myeloid/DC gradients; Treg-rich but skewed	Tissue: multiplex IF-PD-L1 co-localized with CD11c^+^ DCs; fibrosis vs active zones. Blood: sPD-L1 + IgG4	Spatial PD-L1 maps; sPD-L1; EV cargo; IgG4	Steroids/rituximab; consider PD-1/PD-L1 modulation with anti-fibrotic immunometabolic co-therapy
Lerner 2025; Chen 2025 [2,6]	Vitiligo	Insufficient inhibitory tone (Treg number/fitness); metabolic stress	Tissue: perilesional CD8^+^ cytotoxicity; low Tregs. Blood: cytokines; soluble checkpoints; EVs with melanocyte antigens	Treg frequency/forkhead box P3 (FOXP3) stability; IL-17/IL-22; sPD-L1; EV melanocyte antigens	Repigmentation protocols + Treg-boosting (low-dose IL-2) and checkpoint calming; metabolic support for Tregs
Fu 2025; Harel 2025; Kim 2025 [12,15,26]	Cross-cutting (host modifiers)	Immunosenescence/inflammaging; sex differences	Blood: multi-checkpoint PD-1^hi^ phenotypes; reduced naïve pools; sex-stratified cytokines. Tissue: variable ligand display	RAP-style plasma proteomics; sPD-L1; sex-informed panels	Age/sex-aware dosing and vaccine timing; combine metabolic repair, then restore inhibitory tone

Biomarkers and platforms for patient stratification

Tissue and Soluble Checkpoints

Pair lesion mapping with circulation: Tissue immunohistochemistry/immunofluorescence for PD-L1 and second-wave checkpoints with spatial analysis captures compartmental context that blood misses. Pitfalls: ischaemic time, fixation, decalcification, clone variability, epitope loss, and scoring thresholds that may not translate across tissues; importantly, PD-L1 “positivity” may not imply effective inhibition in glycolytic/cytokine-rich niches [2,3]. Soluble sPD-1/sPD-L1/sCTLA-4 in serum/cerebrospinal fluid (CSF) offer dynamic activity/complication readouts; standardize matrix, timing, hemolysis/lipemia handling, freeze-thaw, and platform consistency [27,28].

Exosomes and EVs: EV cargo (proteins/miRNAs/lipids) mirrors checkpoint tone and metabolic state and can include PD-L1, galectin-9, and ICOS-L, yielding composite signatures from blood, saliva, or stool. In systemic autoimmune disease, exosomal miRNAs tied to B-cell activation, Tfh/Tph support, and Treg fragility discriminate activity and organ involvement [6,13]. Methods matter: isolation (ultracentrifugation/size-exclusion/polymer capture), normalization (particle counts vs. tetraspanins), and lipoprotein co-isolation require Minimal Information for Studies of Extracellular Vesicles (MISEV)-style reporting and spike-ins. Engineered exosomes displaying checkpoint agonists or FOXP3-stabilizing miRNAs are emerging co-therapies [23].

Proteomics and multi-omics: Oncology-derived “resistance-associated proteins” (RAPs) panels can stratify autoimmune endotypes where inhibitory signals are present but outpaced by metabolism/cytokines. Blending soluble checkpoints, cytokine modules (IL-21/Tfh), metabolic indicators (lactate dehydrogenase (LDH)/lactate surrogates), and damage signals (4-HNE) can define clusters linked to checkpoint wiring; transcriptomics/epigenomics and EV-omics anchor circulating panels to tissue mechanisms [6,15].

Therapeutic Implications

Therapy works best when it respects sequence and place. Start by reading the tissue: which checkpoint ligands are present, which cells are driving, and what the local chemistry looks like. Then adjust the ground state to lower inflammatory and metabolic noise so inhibitory signals can take hold. Only after the terrain settles should brakes be applied. CTLA-4-Ig remains the simplest way to cool “signal-2.” Dampening CD28-0B7 engagement raises the activation threshold, reduces APC T-cell friction, and lightens the load on downstream checkpoints. It pairs neatly with cytokine control and can be used early in conditions where priming is excessive. The practical effect is a quieter synapse and fewer cells slipping into runaway help. Once co-stimulation is trimmed, smaller doses of other agents tend to work better. PD-1 agonism is a different lever: it enforces tolerance. In joints or glands enriched for PD-1hi Tph/Tfh, the fit is obvious and measurable. Early clinical signals show that gentle activation of this pathway can reduce synovial activity. The approach still needs a disciplined low starting dose, careful staging, infection vigilance, and smart vaccination timing. Track soluble PD-1/PD-L1 or matched tissue readouts, as one can tune and see when the brake is actually gripping.

Not every circuit needs a heavy foot. Second-wave targets LAG-3, TIGIT, BTLA, and VISTA offer context-specific restraint. Small, tissue-aimed combinations can shave Tfh/Tph help and calm follicles without flattening systemic immunity. One practical pattern is to pair a light PD-1 agonist with a second-wave co-signal in the exact organ that misfires. Spatial profiling tells us where that mix belongs; short pharmacokinetic windows keep the touch light. Cells can carry tolerance further. Low-dose IL-2 expands and stabilizes FOXP3+ Tregs, especially in Th17-skewed endotypes. CAR-Tregs add addresses and brakes: antigen-specific, lesion-homing, and built with safety switches so one can pause or redirect. For antibody-heavy disease, B-cell “reset” with anti-CD19 or BCMA CAR-T can debulk autoreactive pools; plan immunoglobulin monitoring, intravenous immunoglobulin (IVIG) support, and infection prophylaxis from day one. Tolerogenic dendritic cells and peptide-coupled cells aim for anergy plus Treg induction while leaving basic defence intact. Sequence still rules: condition first, then deploy cells.

Delivery matters as much as design. Mesenchymal stromal cell-derived or engineered exosomes can ferry small doses of checkpoint agonists, FOXP3-stabilizing microRNAs, or metabolic correctors straight into inflamed niches with modest systemic exposure. Vesicles speak the local “language,” are readily taken up, and can be engineered for homing. What the field needs next is boring but essential: standardized isolation, potency assays, and batch release rules. With that scaffolding, exosome therapeutics fit naturally into a compartment-aware strategy. Immunometabolic co-therapy is the quiet partner that makes everything else land. Lower lactate pressure and lysine lactylation by modulating LDH and MCT1/4 or by targeting the writer/eraser machinery. Trim mTORC1/HIF-1α activity to favor Tregs over Th17/Tfh. Blunt ferroptosis, where peroxidized lipids are driving injury and noise. Run these moves before or in a light overlay with checkpoint restoration. Keep infection guardrails and vaccine scheduling up front. When the chemistry cools, checkpoints gain traction, and small increments deliver outsized effects. Antibody engineering opens two precise lanes beyond “naked” monoclonals. Antibody-drug conjugates can carry immune-re-educating payloads to disease-defining cells, synovial fibroblasts, for instance, so the niche is edited rather than the whole person. Bispecifics can co-engage PD-1 with a regulatory-biased partner, such as an ICOS-Tfr-leaning signal, shaping restraint exactly where needed. Both tools lean on spatial maps to pick targets and on tight PK/PD to avoid systemic drag. Aim small, act local, and measure often.

Putting it together is a practical checklist. Map first: combine tissue immunohistochemistry or immunofluorescence for PD-(L)1 and co-stimulation with soluble checkpoint and EV panels to understand the niche. Condition the terrain: reduce lactate, protect from lipid peroxidation, and quiet NF-κB/JAK-STAT so receptors can work. Apply a primary lever matched to context CTLA-4-Ig for co-stimulation excess; PD-1 agonism for PD-1hi Tph/Tfh synovitis or follicular overdrive; second-wave trims where a softer touch suffices. Reinforce with cells if regulation is fragile: expand Tregs with low-dose IL-2, consider CAR-Tregs in antigen-defined disease, or use B-cell reset with a clear replacement and prophylaxis plan. Deliver precisely to hard-to-reach compartments with engineered exosomes, antibody-drug conjugates, or bispecifics. Monitor and adapt using a composite panel that blends soluble checkpoints, EV miRNAs and ligands, lipid injury markers, and simple clinical anchors, with predefined stop-rules for infection or flare [1,16,18,29-39].

Safety, monitoring, and special populations

Start with infection/malignancy risk: immunosenescence and inflammageing reduce vaccine responses and raise infection susceptibility; schedule influenza/pneumococcal/COVID-19 boosters before deep B- or T-cell “reset” interventions [17,34]. For cell-based tolerance that depletes or reprograms B cells, pre-specify immunoglobulin (Ig) monitoring, prophylaxis, and IVIG rescue for sustained hypogammaglobulinemia [26]. Balance flares vs over-suppression using early-warning panels that combine soluble checkpoints (sPD-L1/sCTLA-4), ferroptosis-linked lipid peroxidation products (e.g., 4-HNE), and EV miRNA/protein signatures; standardize assays before routine use [27]. Sex/pregnancy/aging require explicit protocols: sex-informed dosing and stratification; avoid experimental checkpoint agonists or cellular reprogramming in pregnancy outside specialist oversight/registries; in older adults, integrate checkpoint “tone” with inflammageing indices to prevent iatrogenic immunoparesis [12,26]. For patients with prior oncologic immune checkpoint inhibitor exposure, adapt irAE frameworks and clinical/serologic/tissue signals to guide rebalancing with steroids, targeted immunomodulators, or temporary de-escalation when treating pre-existing autoimmunity [15, 16, 28].

Gaps and controversies

Interpretive Grey Zones

High PD-1 can mark restrained states or active pathology depending on co-signals/metabolism; LAG-3/TIM-3/TIGIT co-expression needs functional assays and spatial context, not binary readouts [4, 15].

Spatial Biology

Endotypes likely arise from evolving micro-niches (PD-L1-rich myeloid foci; Tfh-dense GCs). Port oncology-grade single-cell/spatial-omics to autoimmune lesions with serial sampling to map trajectories and treatment effects [1, 15].

Endotype-First Trials

Practical anchors: Treg-low/Th17-high for Treg support/IL-17 modulation; myeloid-dominant PD-L1-rich for checkpoint agonists/myeloid re-education; lactate-high lesions for metabolic checkpoint combinations [9, 10].

Assay Discipline

Harmonize EV methods and cut-offs; repurpose oncology RAPs panels for multiplex plasma profiling alongside checkpoint agonists or CAR-Tregs. Integrate aging biology, vaccine timing, Tfh quality, and naïve-pool deficits into eligibility, endpoints, and rescue algorithms [13, 28]. Prioritize context-aware safety, build multimodal monitoring (soluble checkpoints + lipid injury signals + EV miRNAs), and run biomarker-anchored, spatially informed trials capable of proving endotype rebalancing.

Critical synthesis

This manuscript reframes autoimmune disease through a crisp, four-fault lens: brake failure, co-stimulation surplus, metabolic/epigenetic drift, and a split between blood and tissue signals. That scaffold is not cosmetic. It explains the recurring paradox of “checkpoint positivity” without clinical restraint and anchors decisions to where pathology lives, the lesion, not the lab slip. The argument is disciplined: biology first, geography second, treatment last. A core strength is the shift from peripheral heuristics to spatial truth. Lesional maps of PD-(L)1 and second-wave receptors, read alongside cellular neighbourhoods, correlate with flare intensity and therapeutic response far better than immune counts in blood. The message is blunt but useful: single markers mislead; composite, compartment-aware panels guide action. Tissue tells us if a brake exists.

Immunometabolism sits at the heart of non-response. Lactate and lysine lactylation push transcription toward inflammatory lineages while loosening FOXP3’s hold on Tregs. Ferroptotic by-products add fuel through NF-κB and stress signalling, raising PD-L1 yet weakening tolerance circuits. In such chemistry, inhibitory receptors can be abundant and still ineffective. The therapeutic corollary is order: condition the niche lower glycolysis, rein in HIF-1α/mTOR, protect membranes before one can press the brake. B-cell help is portrayed with welcome granularity. Tfh/Tph dominance, limp Tfr control, and a hot CD40L/ICOS axis sustain ectopic germinal centres in SLE and Sjögren’s. Abatacept lifts the activation threshold upstream; CD40L antagonism trims germinal centre tempo; gentle PD-1 agonism adds restraint once the fire is cooler. These are not interchangeable levers. They act at different heights of the cascade and should be staged, not stacked blindly.

The biomarker blueprint is pragmatic. Pair lesion immunohistochemistry or spatial IF for PD-(L)1 and second-wave checkpoints with soluble panels (sPD-1, sPD-L1, sCTLA-4) and extracellular-vesicle cargo that mirrors both ligand density and metabolic stress. Pre-analytics are not an afterthought: fixation artefacts, haemolysis, freeze-thaw cycles, and EV isolation bias can swamp signal. Standard operating procedures, not slogans, will make “precision” real. Treatment philosophy is sequence-aware and organ-literate. Start by lowering inflammatory and metabolic noise so inhibitory cues can be heard. Next, cool co-stimulation (CTLA-4-Ig when priming screams) or restore restraint (low-dose PD-1 agonism where PD-1hi Tfh/Tph dominate). Reinforce regulation if fragile low-dose IL-2, and in antigen-defined settings, CAR-Tregs with safety switches. Deliver locally when possible using engineered exosomes, bispecifics, or targeted conjugates to limit systemic drag. Then monitor with a composite panel tied to the original endotype, adjusting early at the first hint of infection or oversuppression.

Disease mosaics are handled without hand-waving. In RA, hypoxia and lipid peroxidation shape a synovium where PD-L1 arrives late and soft; abatacept remains foundational, and metabolic tempering can make later PD-1 signalling meaningful. Sjögren’s is a glandular story of ectopic GCs; CD40L antagonism and IL-21/Tfh trimming fit the architecture, with vesicle therapies aiming to tutor the tissue. SLE requires Treg stabilisation and B-cell resets first; any checkpoint agonism should be light and endotype-bound. Psoriasis/PsA follows a “fuel before flame” logic: lower lactate and reactive lipids, then consider restraint. In MS, gates matter; prepare the barrier and myeloid ligands before any attempt to deepen brakes. T1D highlights β-cell stress with paradoxical PD-L1 upregulation; anti-inflammatory conditioning and Treg support precede precision tolerance. IBD demands mucosal endotyping with ferroptosis and lactate markers; only subsets with amenable chemistry should see checkpoint calming. AAV, IgG4-RD, and vitiligo each get a compact, place-specific plan rather than generic suppression. Safety is treated with the seriousness it deserves. Vaccinate before deep B- or T-cell edits. Track immunoglobulins when depleting B cells and keep IVIG plans explicit. Use early-warning composites, soluble checkpoints, ferroptosis-linked lipids, and EV miRNAs, rather than waiting for a clinical cliff. Ageing and sex are not footnotes: smaller naïve pools, PD-1hi profiles, and hormone pathway crosstalk change dose needs and infection risk. Pregnancy and prior checkpoint-inhibitor exposure warrant specialist protocols and conservative thresholds.

The manuscript is candid about frictions. Spatial and EV platforms are unevenly available; cut-down surrogate panels should be specified for centres without advanced assays. Extracellular-vesicle methods remain fragmented; without harmonised isolation, normalisation, and potency testing, EV signatures risk irreproducibility. Dose, schedule, and sequence for PD-1 or second-wave agonism in autoimmunity are early-stage; organ-targeted, small adaptive trials with tight pharmacodynamic readouts are the right next step. Trial design recommendations feel practical, not aspirational. Recruit by endotype Treg-low/Th17-high, PD-L1-rich myeloid, lactate-high mucosa rather than by umbrella label alone. Make metabolic conditioning part of the randomised package, not an optional prelude. Pair disease activity endpoints with infection and immunoparesis composites. Use adaptive dosing steered by soluble/EV checkpoints and lipid injury markers. Reserve platform arms for delivery tech-engineered exosomes, bispecifics, and antibody-drug conjugates, so the assay core and safety playbook stay constant while modalities iterate.

Implementation can scale. A minimal starter kit looks like this: one targeted biopsy with PD-L1 plus a second-wave receptor scored under a fixed SOP; one blood draw with sPD-1/sPD-L1/sCTLA-4, a small cytokine module, LDH as a metabolic proxy, and a basic EV panel run to a locked protocol. Conditions with available agents that lower glycolysis or lipid peroxidation without blunting antimicrobial defence. If co-stimulation is loud, start abatacept. If PD-1hi Tfh/Tph circuits dominate in a cooled niche, consider low-dose PD-1 agonism within a monitored pathway. Add low-dose IL-2 when Treg fragility is documented.

## Conclusions

Immune checkpoints function as graded rheostats, and autoimmune pathology most often reflects four overlapping faults: loss of inhibitory tone, excess co-stimulation, metabolic/epigenetic decoupling, and tissue blood mismatch. Stratification should be tissue-anchored and paired with soluble and extracellular-vesicle markers, interpreted under strict assay discipline. Therapy ought to be sequence-aware: first lower inflammatory and metabolic load, then restore inhibitory tone with checkpoint agonists or co-stimulation blockade, complemented by Treg-centered strategies and EV-guided delivery where appropriate. Safety requires planned vaccine timing, age- and sex-aware dosing, and composite early-warning panels that distinguish flare from over-suppression. The priority now is endotype-first, spatially informed, biomarker-anchored trials capable of delivering durable, safer immune rebalancing.
